# FK228 from *Burkholderia thailandensis* MSMB43

**DOI:** 10.1107/S160053681203601X

**Published:** 2012-08-23

**Authors:** Xiang-Yang Liu, Cheng Wang, Yi-Qiang Cheng

**Affiliations:** a Department of Biological Sciences, Department of Chemistry and Biochemistry, Univeristy of Wisconsin–Milwaukee, PO Box 413, Milwaukee, WI 53201, USA

## Abstract

FK228 [systematic name: (1*S*,4*S*,7*Z*,10*S*,16*E*,21*R*)-7-ethyl­idene-4,21-di(propan-2-yl)-2-oxa-12,13-dithia-5,8,20,23-tetra­za­­bicyclo[8.7.6]tricos-16-ene-3,6,9,19,22-pentone], C_24_H_36_N_4_O_6_S_2_, also known as FR901228, depsipeptide, NSC 630176, romidepsin, and marketed as Istodax by Celgene Corporation, is crystallized from ethyl acetate in *P*2_1_ as compared to the absolute configuration of FK228, first crystallized from methanol in *P*2_1_2_1_2_1_ [Shigematsu *et al.* (1994 ▶). *J. Anti­biot.* **47**, 311–314]. A slight difference is observed between the absolute configuration of FK228 and the present structure. The molecular structure is stabilized by intramolecular N—H⋯O hydrogen bonds. In the crystal, molecules are linked *via* N—H⋯O hydrogen bonds.

## Related literature

For diverse natural products, see: Nguyen *et al.* (2008 ▶); Knappe *et al.* (2008 ▶); Seyedsayamdost *et al.* (2010 ▶); Biggins *et al.* (2011 ▶); Klausmeyer *et al.* (2011 ▶); Wang *et al.* (2011 ▶, 2012 ▶). For large-scale genome sequencing, see: Yu *et al.* (2006 ▶); Mukhopadhyay *et al.* (2010 ▶); Zhuo *et al.* (2012 ▶). For the initial discovery of FK228 from *Chromobacterium violaceum* No. 968 and its crystal structure report, see: Shigematsu *et al.* (1994 ▶); Ueda, Nakajima, Hori, Fujita *et al.* (1994 ▶). For the biological activities and mode of action of FK228, see: Furumai *et al.* (2002 ▶); Ueda, Manda *et al.* (1994 ▶); Ueda, Nakajima, Hori, Goto & Okuhara (1994 ▶). For biosynthetic studies of FK228, see: Cheng *et al.* (2007 ▶); Potharla *et al.* (2011 ▶); Wesener *et al.* (2011 ▶). For clinical application of FK228, see: Robey *et al.* (2011 ▶); StatBite (2010 ▶).
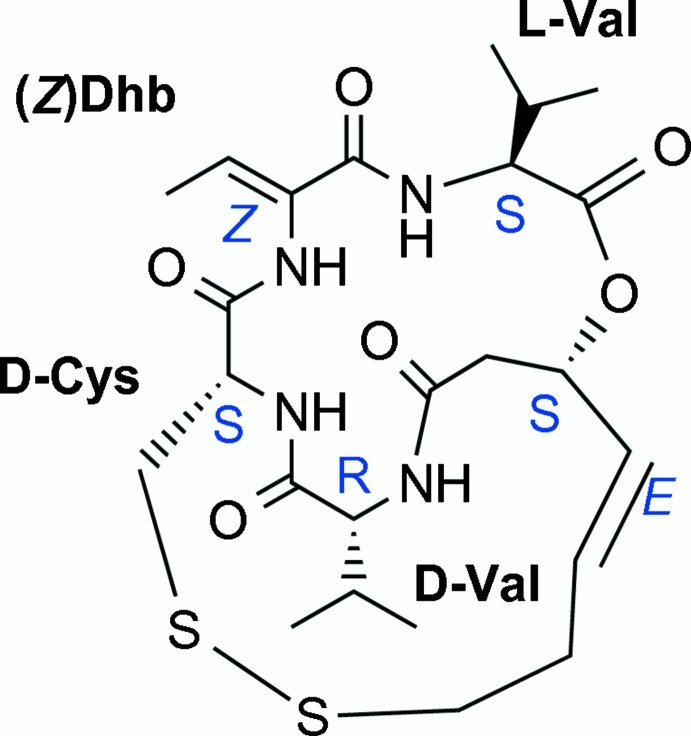


## Experimental

### 

#### Crystal data

C_24_H_36_N_4_O_6_S_2_
*M* *_r_* = 540.69
Monoclinic, 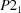

*a* = 9.1085 (2) Å
*b* = 16.2431 (4) Å
*c* = 9.4192 (2) Å
β = 92.096 (1)°
*V* = 1392.64 (5) Å^3^
*Z* = 2
Cu *K*α radiation
μ = 2.10 mm^−1^
*T* = 100 K
0.26 × 0.14 × 0.07 mm

#### Data collection

Bruker SMART APEXII area-detector diffractometer
Absorption correction: analytical (*SADABS*; Sheldrick, 2003 ▶) *T*_min_ = 0.611, *T*_max_ = 0.867
22577 measured reflections
4918 independent reflections
4859 reflections with *I* > 2σ(*I*)
*R*_int_ = 0.025

#### Refinement

*R*[*F* ^2^ > 2σ(*F* ^2^)] = 0.025
*wR*(*F* ^2^) = 0.065
*S* = 1.02
4918 reflections
343 parameters
1 restraint
H atoms treated by a mixture of independent and constrained refinement
Δρ_max_ = 0.29 e Å^−3^
Δρ_min_ = −0.15 e Å^−3^
Absolute structure: Flack (1983 ▶), 2188 Friedel pairs
Flack parameter: 0.022 (9)

### 

Data collection: *APEX2* (Bruker, 2007 ▶); cell refinement: *SAINT* (Bruker, 2007 ▶); data reduction: *SAINT*; program(s) used to solve structure: *SHELXTL* (Sheldrick, 2008 ▶); program(s) used to refine structure: *SHELXTL*; molecular graphics: *SHELXTL* and *OLEX2* (Dolomanov *et al.*, 2009 ▶); software used to prepare material for publication: *SHELXTL*.

## Supplementary Material

Crystal structure: contains datablock(s) global, I. DOI: 10.1107/S160053681203601X/jj2148sup1.cif

Structure factors: contains datablock(s) I. DOI: 10.1107/S160053681203601X/jj2148Isup2.hkl

Supplementary material file. DOI: 10.1107/S160053681203601X/jj2148Isup3.cdx

Additional supplementary materials:  crystallographic information; 3D view; checkCIF report

## Figures and Tables

**Table 1:** Hydrogen-bond geometry (Å, °)

*D*—H⋯*A*	*D*—H	H⋯*A*	*D*⋯*A*	*D*—H⋯*A*
N1—H3⋯O5	0.84 (2)	2.27 (2)	3.0123 (18)	147.1 (17)
N2—H4⋯O6	0.82 (2)	2.05 (2)	2.7867 (18)	149.1 (18)
N3—H16⋯O3^i^	0.87 (2)	2.18 (2)	3.0449 (17)	175.9 (18)

Symmetry code: (i) 
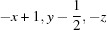
.

**Fig. 1.**
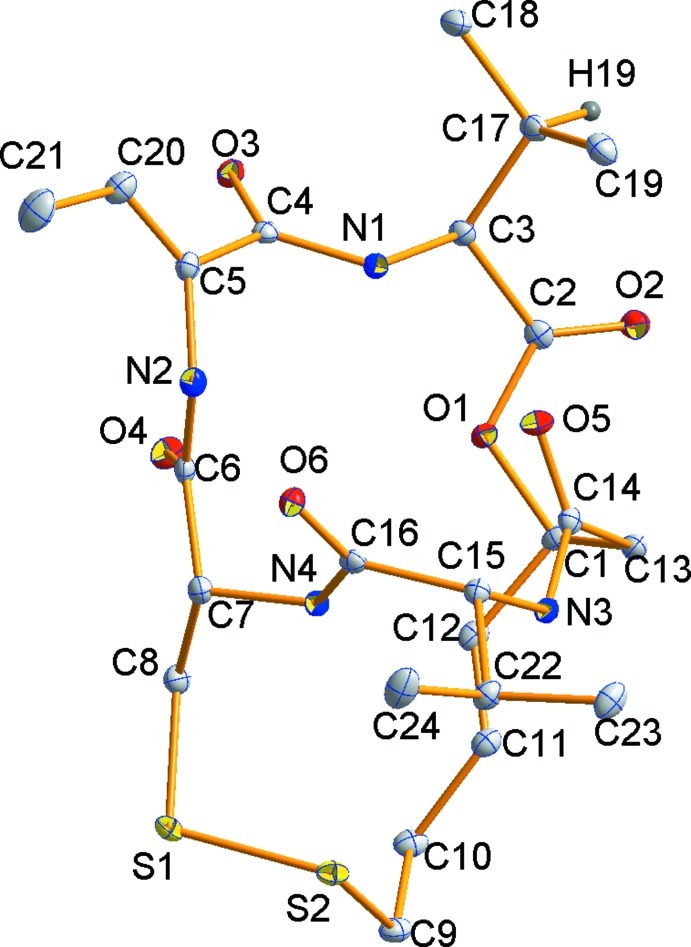
A molecular structure of FK228 with displacement ellipsoids shown at the 30% probability level. All hydrogen atoms were omitted for clarity except for H19.

**Fig. 2.**
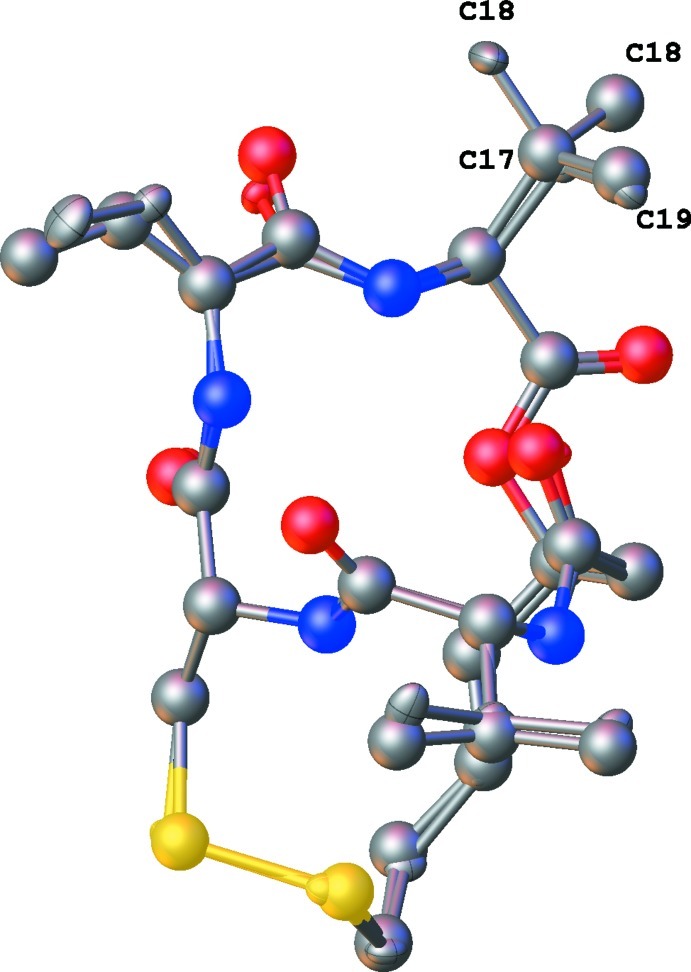
Overlaid crystal structures of previous (isolated from Chromobacterium violaceum No. 968) (Balls & sticks) and newly (Ellipses & sticks) isolated FK228.

**Fig. 3.**
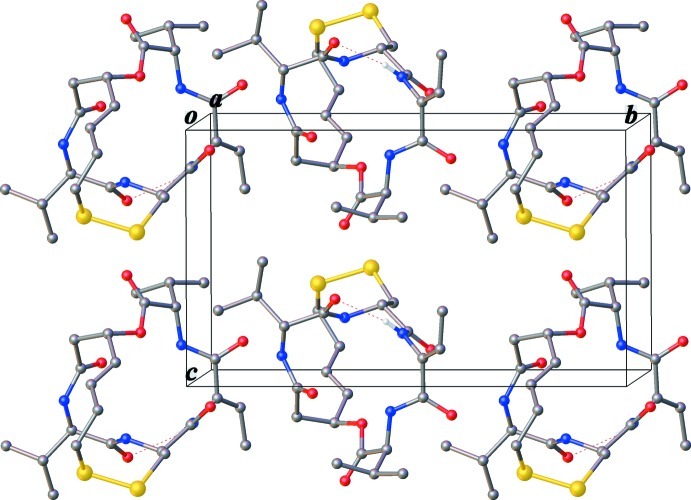
A packing diagram of FK228, viewed along the a axis. The dashed lines represent N—H···O hydrogen bonds.

## supplementary crystallographic information

### Comment

Diverse natural products, including thailandamides (Nguyen *et al.*, 2008), capistruin (Knappe *et al.*, 2008), bactobolin A—D (Seyedsayamdost *et al.*, 2010), burkholdacs A—B (Biggins *et al.*, 2011), spiruchostatin C (Klausmeyer *et al.*, 2011), and thailandepsins (Wang *et al.*, 2011, 2012), were discovered recently from *Brukholderia thailandensis* E264. In conjunction with large-scale genome sequencing (Yu *et al.*, 2006; Mukhopadhyay *et al.*, 2010; Zhuo *et al.*, 2012), the *Burkholderia thailandensis* species have drawn much attention due to their capability to synthesize novel compounds with antibacterial, antitumor and antiviral activities.

While exploring novel natural products from the culture broth of *B. thailandensis* MSMB43, the title compound FK228 was isolated in large quantity (~100 mg/*L*). The physicochemical properties (UV spectrum, IR spectrum, MS and NMR) of the FK228 from *B. thailandensis* MSMB43 are identical to those of the FK228 isolated in the early 1990s from *Chromobacterium violaceum* No. 968 (Ueda, Nakajima, Hori, Fujita *et al.*, 1994; Shigematsu *et al.*, 1994). The crystal structure of the newly isolated FK228, herein, is slightly different than that reported by (Ueda, Manda *et al.*, 1994), due to different crystallization conditions employed.

FK228 is bicyclic depsipeptide and consists of five building blocks, *D*-cysteine (*D*-Cys), *D*-valine (*D*-Val), 4-amino-3-hydroxy-5-methylheptanoic acid (Ahhp, derived from an isoleucine and an acetate unit), *L*-valine (*L*-Val), *Z*-dehydrobutyrine(*Z-*Dhb) and 3-hydroxy-7-mercapto-4-heptenoic acid (Acyl, derived from a cysteine and two acetate units). The primary structure of FK228 is (*L*)Val-(*Z*)Dhb-(*D*)Cys-Ahhp-(*D*)Val. X-ray crystallographic analysis indicates that the skeleton of FK228 is a [8.7.6] 23-membered ring adopting an uncommon cage-shape including a 16-membered macrocyclic lactone and a 15-membered ring containing a signature disulfide bond.

Under the different crystallization conditions presented, FK228 formed a different molecular arrangement than previously described (Fig. 1). The title crystal structure was obtained from ethyl acetate crystallizing in the *P*2_1_ space group, while the provirus structure co-crystallized with one methanol in asymmetric unit in the *P*2_1_2_1_2_1_ space group. The absolute configurations of all the chiral centers in each of these two structures are the same: C1(*S*), C3(*S*), c7 (*S*) and C22 (*R*). The geometric configuration of the double bonds in the Acyl and Dhb components are all determined as *E* and *Z* respectively. In comparing the these two structures, the configuration of these skeletons are the same. But there is a slight difference at the end of *L*-Val group. Under the different crystallization conditions, C18 and H19 had the opposite positions in these two structures because of the free rotation of the single bond (Fig. 2). Hydrogen bonds N2—H4···O6 and the weak intermolecular interactions N1—H3···O5 and N3—H16···O3 are observed in the title crystal structure. (Table 1, Fig. 3).

### Experimental

Bacteria and culture medium

*Burkholderia thailandensis* strain MSMB43 (obtained from the US Centers for Disease Control, CDC) was routinely activated on LB agar containing 50 mg ml^-1^ of apramycin (Am50) at 37°C for 1 to 2 days as a master plate. A single colony was then transferred into a 1 *L* flask containing 300 ml of LB medium and Am50, and the culture were growing at 37°C for 24 h as seed culture. For batch fermentation 250 ml of seed culture was transferred into a 20 *L* fermentor (BioFlo IV, New Brunswick Scientific Co., USA) containing 12 *L* of M8 medium (0.5% glucose, 0.5% peptone, 0.3% NaCl, 0.12% Na_2_HPO_4_, and 0.05% KH_2_PO_4_; pH 7), and fermented at 30°C for 96 hr under 20 *L*/min aeration and 200 rpm agitation; pH was maintained at 7.0 with 1 N NaCl or 1M HCl. For fed-batch fermentation, feeding of 3 *L* of 10X concentrated medium between 24 hr and 48 hr was performed on top of the batch fermentation.

Recovery of the crude extract

Bacteria cells were removed by centrifugation of cell broth at 3,800 rpm for 15 min. The supernatant was applied on a 2 *L* column (*Φ* 8.0 × 40 cm) packed with a mixture of Diaion HP-20 (Sigma-Aldrich, USA) resin and Amberlite XAD16 (Sigma-Aldrich, USA), which has been equilibrated in water at 5 ml/min. The resin was dried and then extracted with ethyl acetate for three times. The ethyl acetate extracts were combined and concentrated to dryness *in vacuo* at 35°C.

Isolation and purification of the title compound

The ethyl acetate extract was subjected to a two step isolation and purification protocol. Briefly, the ethyl acetate extract was mixed with 50 g silica gel (230–400 mesh, Whatman Purasil, USA). The mixture of silica gel was dried overnight and then applied to a 120 - g silica gel column (300 ml), which has been equilibrated with hexane. The column was eluted sequentially with 1 *L* hexane, 1 *L* hexane: ethyl acetate (3:1, *v*/*v*), 1 *L* hexane: ethyl acetate (1:1, *v*/*v*), 1 *L* ethyl acetate, 1 *L* ethyl acetate: acetone (1:1, *v*/*v*), and finally 2 *L* of acetone. The obtained fractions were applied on a flash chromatography equipped with a silica gel universal column (Yamazen Corporation, 23 ×123 mm, 16 g) connected to an injection column (Yamazen Corporation, 20 × 65 mm, 14 g). The column was eluted by a mixture of chloroform and acetone with an increasing of polarity according to the following concentrations of acetone, 1%, 5%, 10%, 15%, 20%, 25%, 30%, 35%, 65%, and 100%. Fractions by 15% and 20% acetone were concentrated into dryness *in vacuo* and dissolved in acetonitrile. The acetonitrile solution was applied on a preparative HPLC system equipped with an Agilent Prep-C18 column (21.2 × 250 mm, 10 µm, PN 410910–102, USA). The mobile phase was composed of acetonitrile and water (purified by NANO pure Diamond Life Science ultrapure water system). After loading the sample, the column was eluted by a linear gradient aqueous acetonitrile from 40% to 55% within 0–25 min, FK228 was collected at 21–25 min. The flow rate was 8 ml/min. The UV spectrum was monitored at 210 nm. The FK228 solution was concentrated into dryness *in vacuo* on a rotary evaporator at 35 °C. The dried FK228 was dissolved in ethyl acetate and the crystals were obtained at room temperature after 5–7 days.

### Refinement

All hydrogen atoms attached to the carbon atoms were placed in geometrically idealized positions (C—H = 0.98, 0.99 and 1.00 Å on the primary, secondary and tertiary aliphatic C atoms respectively, 0.95 Å on aromatic C). The H atoms were refined as riding, with isotropic displacement coefficients of *U*iso(H) = 1.5*U*eq(C) for methyl groups or 1.2*U*eq(C) otherwise. The hydrogen atoms attached to N and O were located in difference maps and refined independently with restraints and constraints. The H atoms on N atoms were restrained to have N—H distances of 0.880 (1) Å and their *U*iso values were constrained as equal to 1.2 times the *U*eq of their carrier atoms. The H atom on O was restrained to have an O—H distance of 0.840 (1) Å and the *U*iso value was assigned as equal to 1.5 times the *U*eq of the oxygen atom.

### Crystal data

**Table:**

C_24_H_36_N_4_O_6_S_2_	*F*(000) = 576
*M**_r_* = 540.69	*D*_x_ = 1.289 Mg m^−^^3^
Monoclinic, *P*2_1_	Cu *K*α radiation, λ = 1.54178 Å
Hall symbol: P 2yb	Cell parameters from 999 reflections
*a* = 9.1085 (2) Å	θ = 4.7–69.8°
*b* = 16.2431 (4) Å	µ = 2.10 mm^−^^1^
*c* = 9.4192 (2) Å	*T* = 100 K
β = 92.096 (1)°	Block, colourless
*V* = 1392.64 (5) Å^3^	0.26 × 0.14 × 0.07 mm
*Z* = 2	

### Data collection

**Table:**

Bruker SMART APEXII area-detector diffractometer	4918 independent reflections
Radiation source: fine-focus sealed tube	4859 reflections with *I* > 2σ(*I*)
Graphite monochromator	*R*_int_ = 0.025
0.50° ω and 0.5 ° φ scans	θ_max_ = 69.8°, θ_min_ = 4.7°
Absorption correction: analytical (*SADABS*; Sheldrick, 2003)	*h* = −10→11
*T*_min_ = 0.611, *T*_max_ = 0.867	*k* = −19→19
22577 measured reflections	*l* = −11→11

### Refinement

**Table:**

Refinement on *F*^2^	Secondary atom site location: difference Fourier map
Least-squares matrix: full	Hydrogen site location: inferred from neighbouring sites
*R*[*F*^2^ > 2σ(*F*^2^)] = 0.025	H atoms treated by a mixture of independent and constrained refinement
*wR*(*F*^2^) = 0.065	*w* = 1/[σ^2^(*F*_o_^2^) + (0.0447*P*)^2^ + 0.2408*P*] where *P* = (*F*_o_^2^ + 2*F*_c_^2^)/3
*S* = 1.02	(Δ/σ)_max_ = 0.001
4918 reflections	Δρ_max_ = 0.29 e Å^−^^3^
343 parameters	Δρ_min_ = −0.15 e Å^−^^3^
1 restraint	Absolute structure: Flack (1983), 2188 Friedel pairs
Primary atom site location: structure-invariant direct methods	Flack parameter: 0.022 (9)

### Special details

**Table:**

Geometry. All e.s.d.'s (except the e.s.d. in the dihedral angle between two l.s. planes) are estimated using the full covariance matrix. The cell e.s.d.'s are taken into account individually in the estimation of e.s.d.'s in distances, angles and torsion angles; correlations between e.s.d.'s in cell parameters are only used when they are defined by crystal symmetry. An approximate (isotropic) treatment of cell e.s.d.'s is used for estimating e.s.d.'s involving l.s. planes.
Refinement. Refinement of *F*^2^ against ALL reflections. The weighted *R*-factor *wR* and goodness of fit *S* are based on *F*^2^, conventional *R*-factors *R* are based on *F*, with *F* set to zero for negative *F*^2^. The threshold expression of *F*^2^ > σ(*F*^2^) is used only for calculating *R*-factors(gt) *etc*. and is not relevant to the choice of reflections for refinement. *R*-factors based on *F*^2^ are statistically about twice as large as those based on *F*, and *R*- factors based on ALL data will be even larger.

### Fractional atomic coordinates and isotropic or equivalent isotropic displacement parameters (Å^2^)

**Table:**

	*x*	*y*	*z*	*U*_iso_*/*U*_eq_	
S1	0.21741 (4)	0.87614 (3)	0.41260 (4)	0.02391 (9)	
S2	0.21966 (4)	0.75561 (3)	0.35057 (4)	0.02177 (9)	
O1	0.47759 (11)	0.86900 (7)	−0.17912 (11)	0.0166 (2)	
O2	0.54893 (12)	0.83426 (7)	−0.39718 (11)	0.0228 (2)	
O3	0.73140 (12)	1.08712 (7)	−0.12864 (11)	0.0188 (2)	
O4	0.48889 (12)	1.02648 (7)	0.11054 (12)	0.0234 (2)	
O5	0.68455 (11)	0.76827 (7)	−0.04745 (11)	0.0206 (2)	
O6	0.74839 (11)	0.82368 (7)	0.32442 (11)	0.0175 (2)	
N1	0.72350 (14)	0.94804 (8)	−0.11503 (13)	0.0155 (2)	
H3	0.724 (2)	0.9060 (13)	−0.063 (2)	0.019*	
N2	0.70812 (14)	0.97489 (8)	0.18875 (13)	0.0170 (3)	
H4	0.750 (2)	0.9394 (13)	0.237 (2)	0.020*	
N3	0.54236 (14)	0.69120 (8)	0.09041 (13)	0.0153 (2)	
H16	0.467 (2)	0.6595 (13)	0.099 (2)	0.018*	
N4	0.51449 (14)	0.83271 (8)	0.23715 (14)	0.0159 (3)	
H18	0.454 (2)	0.8103 (13)	0.195 (2)	0.019*	
C1	0.37318 (16)	0.80077 (9)	−0.18177 (16)	0.0171 (3)	
H1	0.3247	0.7979	−0.2786	0.021*	
C2	0.56600 (16)	0.87327 (10)	−0.28962 (14)	0.0168 (3)	
C3	0.68786 (16)	0.93675 (10)	−0.26594 (15)	0.0167 (3)	
H2	0.6488	0.9905	−0.3027	0.020*	
C4	0.74312 (15)	1.02321 (10)	−0.05887 (15)	0.0157 (3)	
C5	0.78862 (16)	1.02503 (9)	0.09680 (15)	0.0172 (3)	
C6	0.55899 (17)	0.97821 (9)	0.18570 (15)	0.0169 (3)	
C7	0.48646 (16)	0.91621 (10)	0.28501 (15)	0.0171 (3)	
H5A	0.5281	0.9234	0.3839	0.021*	
C8	0.32175 (17)	0.93220 (10)	0.28278 (16)	0.0200 (3)	
H6	0.2808	0.9185	0.1869	0.024*	
H7	0.3061	0.9918	0.2977	0.024*	
C9	0.05712 (16)	0.74578 (10)	0.23338 (17)	0.0216 (3)	
H8	0.0435	0.6869	0.2092	0.026*	
H9	−0.0295	0.7635	0.2860	0.026*	
C10	0.06058 (16)	0.79491 (11)	0.09558 (17)	0.0214 (3)	
H10	−0.0326	0.7857	0.0404	0.026*	
H11	0.0667	0.8543	0.1189	0.026*	
C11	0.18736 (16)	0.77249 (10)	0.00426 (16)	0.0190 (3)	
H12	0.2182	0.7166	0.0052	0.023*	
C12	0.25928 (16)	0.82409 (10)	−0.07712 (17)	0.0190 (3)	
H13	0.2369	0.8810	−0.0687	0.023*	
C13	0.45645 (17)	0.71982 (10)	−0.15341 (15)	0.0172 (3)	
H14	0.3854	0.6764	−0.1291	0.021*	
H15	0.5048	0.7027	−0.2411	0.021*	
C14	0.57163 (16)	0.72764 (9)	−0.03355 (15)	0.0162 (3)	
C15	0.64392 (16)	0.70259 (9)	0.21230 (15)	0.0154 (3)	
H17	0.7451	0.6916	0.1792	0.018*	
C16	0.64070 (16)	0.79203 (9)	0.26422 (14)	0.0153 (3)	
C17	0.82380 (17)	0.91592 (10)	−0.35219 (16)	0.0199 (3)	
H19	0.7900	0.9098	−0.4539	0.024*	
C18	0.93314 (19)	0.98732 (11)	−0.34389 (17)	0.0274 (4)	
H22	0.8836	1.0383	−0.3742	0.041*	
H21	1.0145	0.9761	−0.4063	0.041*	
H20	0.9714	0.9934	−0.2459	0.041*	
C19	0.89729 (19)	0.83572 (11)	−0.30681 (18)	0.0257 (4)	
H24	0.9370	0.8410	−0.2091	0.039*	
H25	0.9773	0.8234	−0.3703	0.039*	
H23	0.8250	0.7910	−0.3118	0.039*	
C20	0.90281 (18)	1.07072 (10)	0.14031 (17)	0.0230 (3)	
H26	0.9536	1.1000	0.0699	0.028*	
C21	0.9578 (2)	1.07966 (13)	0.2911 (2)	0.0358 (4)	
H27	0.8802	1.0637	0.3550	0.054*	
H29	0.9856	1.1371	0.3093	0.054*	
H28	1.0436	1.0441	0.3079	0.054*	
C22	0.61246 (18)	0.63859 (10)	0.32827 (17)	0.0201 (3)	
H30	0.5052	0.6402	0.3475	0.024*	
C23	0.65029 (19)	0.55289 (10)	0.27385 (19)	0.0253 (3)	
H31	0.5935	0.5418	0.1855	0.038*	
H32	0.6262	0.5117	0.3453	0.038*	
H33	0.7555	0.5502	0.2558	0.038*	
C24	0.6992 (2)	0.65666 (11)	0.46668 (18)	0.0301 (4)	
H35	0.8045	0.6571	0.4488	0.045*	
H34	0.6786	0.6140	0.5368	0.045*	
H36	0.6700	0.7105	0.5033	0.045*	

### Atomic displacement parameters (Å^2^)

**Table:**

	*U*^11^	*U*^22^	*U*^33^	*U*^12^	*U*^13^	*U*^23^
S1	0.02086 (19)	0.0278 (2)	0.02363 (18)	0.00080 (16)	0.00810 (13)	−0.00407 (16)
S2	0.01743 (17)	0.02238 (19)	0.02566 (19)	0.00194 (15)	0.00309 (13)	0.00550 (15)
O1	0.0174 (5)	0.0153 (5)	0.0171 (5)	−0.0017 (4)	0.0011 (4)	0.0016 (4)
O2	0.0244 (6)	0.0277 (6)	0.0161 (5)	−0.0058 (5)	−0.0005 (4)	−0.0013 (5)
O3	0.0221 (5)	0.0140 (5)	0.0204 (5)	−0.0002 (4)	0.0005 (4)	0.0020 (4)
O4	0.0225 (6)	0.0182 (6)	0.0295 (6)	0.0029 (4)	−0.0013 (5)	0.0046 (5)
O5	0.0159 (5)	0.0223 (6)	0.0236 (5)	−0.0028 (4)	0.0004 (4)	0.0032 (4)
O6	0.0171 (5)	0.0176 (5)	0.0175 (5)	0.0001 (4)	−0.0034 (4)	−0.0004 (4)
N1	0.0191 (6)	0.0142 (6)	0.0132 (6)	−0.0004 (5)	0.0006 (5)	0.0013 (5)
N2	0.0199 (6)	0.0152 (6)	0.0157 (6)	0.0017 (5)	−0.0022 (5)	0.0012 (5)
N3	0.0141 (6)	0.0140 (6)	0.0179 (6)	−0.0013 (5)	0.0000 (5)	−0.0006 (5)
N4	0.0139 (6)	0.0150 (6)	0.0187 (6)	−0.0002 (5)	−0.0011 (5)	−0.0017 (5)
C1	0.0168 (7)	0.0170 (8)	0.0174 (7)	−0.0040 (6)	−0.0028 (5)	0.0020 (6)
C2	0.0189 (7)	0.0183 (7)	0.0130 (6)	0.0034 (6)	−0.0021 (5)	0.0040 (6)
C3	0.0181 (7)	0.0177 (7)	0.0144 (6)	−0.0005 (6)	−0.0001 (5)	0.0015 (6)
C4	0.0111 (6)	0.0171 (7)	0.0190 (7)	−0.0007 (5)	0.0030 (5)	0.0001 (6)
C5	0.0182 (7)	0.0141 (7)	0.0192 (7)	0.0018 (6)	0.0004 (5)	−0.0011 (6)
C6	0.0206 (7)	0.0128 (7)	0.0172 (7)	0.0004 (6)	0.0010 (6)	−0.0048 (6)
C7	0.0184 (7)	0.0171 (7)	0.0159 (7)	0.0006 (6)	0.0010 (5)	−0.0021 (6)
C8	0.0196 (7)	0.0167 (7)	0.0238 (7)	0.0023 (6)	0.0033 (6)	−0.0011 (6)
C9	0.0143 (7)	0.0229 (9)	0.0278 (8)	−0.0014 (6)	0.0033 (6)	0.0025 (7)
C10	0.0146 (7)	0.0220 (8)	0.0275 (8)	0.0015 (6)	−0.0007 (6)	0.0041 (7)
C11	0.0163 (7)	0.0173 (8)	0.0231 (7)	0.0001 (6)	−0.0024 (6)	0.0012 (6)
C12	0.0159 (7)	0.0161 (7)	0.0246 (8)	0.0016 (6)	−0.0038 (6)	0.0022 (6)
C13	0.0189 (7)	0.0170 (7)	0.0157 (7)	−0.0014 (6)	0.0013 (5)	−0.0001 (6)
C14	0.0166 (7)	0.0135 (7)	0.0185 (7)	0.0027 (6)	0.0010 (5)	−0.0012 (6)
C15	0.0127 (7)	0.0160 (7)	0.0173 (7)	0.0008 (5)	−0.0007 (5)	−0.0003 (6)
C16	0.0164 (7)	0.0181 (7)	0.0114 (6)	−0.0009 (6)	0.0014 (5)	0.0014 (6)
C17	0.0196 (7)	0.0263 (8)	0.0141 (6)	−0.0025 (7)	0.0017 (5)	−0.0025 (6)
C18	0.0245 (8)	0.0333 (10)	0.0249 (8)	−0.0088 (7)	0.0087 (7)	−0.0060 (7)
C19	0.0219 (8)	0.0319 (9)	0.0235 (8)	0.0026 (7)	0.0035 (6)	−0.0028 (7)
C20	0.0228 (8)	0.0198 (8)	0.0259 (8)	−0.0017 (6)	−0.0038 (6)	0.0025 (6)
C21	0.0375 (10)	0.0349 (11)	0.0338 (10)	−0.0069 (8)	−0.0157 (8)	−0.0019 (8)
C22	0.0214 (8)	0.0171 (8)	0.0215 (7)	−0.0006 (6)	−0.0021 (6)	0.0021 (6)
C23	0.0248 (8)	0.0166 (8)	0.0340 (9)	0.0021 (6)	−0.0072 (7)	0.0032 (7)
C24	0.0458 (11)	0.0210 (9)	0.0226 (8)	−0.0021 (8)	−0.0097 (7)	0.0059 (7)

### Geometric parameters (Å, º)

**Table:**

S1—C8	1.8200 (16)	C9—H9	0.9900
S1—S2	2.0434 (6)	C10—C11	1.510 (2)
S2—C9	1.8211 (15)	C10—H10	0.9900
O1—C2	1.3408 (17)	C10—H11	0.9900
O1—C1	1.4599 (18)	C11—C12	1.325 (2)
O2—C2	1.2003 (19)	C11—H12	0.9500
O3—C4	1.2311 (19)	C12—H13	0.9500
O4—C6	1.2208 (19)	C13—C14	1.518 (2)
O5—C14	1.2331 (19)	C13—H14	0.9900
O6—C16	1.2277 (19)	C13—H15	0.9900
N1—C4	1.340 (2)	C15—C16	1.534 (2)
N1—C3	1.4580 (18)	C15—C22	1.542 (2)
N1—H3	0.84 (2)	C15—H17	1.0000
N2—C6	1.359 (2)	C17—C19	1.519 (2)
N2—C5	1.413 (2)	C17—C18	1.529 (2)
N2—H4	0.82 (2)	C17—H19	1.0000
N3—C14	1.344 (2)	C18—H22	0.9800
N3—C15	1.4596 (18)	C18—H21	0.9800
N3—H16	0.87 (2)	C18—H20	0.9800
N4—C16	1.342 (2)	C19—H24	0.9800
N4—C7	1.455 (2)	C19—H25	0.9800
N4—H18	0.76 (2)	C19—H23	0.9800
C1—C12	1.505 (2)	C20—C21	1.496 (2)
C1—C13	1.537 (2)	C20—H26	0.9500
C1—H1	1.0000	C21—H27	0.9800
C2—C3	1.525 (2)	C21—H29	0.9800
C3—C17	1.543 (2)	C21—H28	0.9800
C3—H2	1.0000	C22—C23	1.527 (2)
C4—C5	1.509 (2)	C22—C24	1.528 (2)
C5—C20	1.330 (2)	C22—H30	1.0000
C6—C7	1.540 (2)	C23—H31	0.9800
C7—C8	1.522 (2)	C23—H32	0.9800
C7—H5A	1.0000	C23—H33	0.9800
C8—H6	0.9900	C24—H35	0.9800
C8—H7	0.9900	C24—H34	0.9800
C9—C10	1.525 (2)	C24—H36	0.9800
C9—H8	0.9900		
			
C8—S1—S2	106.04 (5)	C11—C12—C1	125.93 (15)
C9—S2—S1	103.94 (6)	C11—C12—H13	117.0
C2—O1—C1	115.81 (12)	C1—C12—H13	117.0
C4—N1—C3	121.41 (13)	C14—C13—C1	112.39 (13)
C4—N1—H3	120.8 (13)	C14—C13—H14	109.1
C3—N1—H3	117.6 (13)	C1—C13—H14	109.1
C6—N2—C5	120.32 (13)	C14—C13—H15	109.1
C6—N2—H4	118.8 (14)	C1—C13—H15	109.1
C5—N2—H4	120.4 (14)	H14—C13—H15	107.9
C14—N3—C15	119.16 (13)	O5—C14—N3	121.37 (13)
C14—N3—H16	122.0 (13)	O5—C14—C13	121.48 (13)
C15—N3—H16	118.8 (13)	N3—C14—C13	117.09 (13)
C16—N4—C7	123.97 (13)	N3—C15—C16	110.61 (12)
C16—N4—H18	117.8 (15)	N3—C15—C22	110.05 (12)
C7—N4—H18	118.2 (15)	C16—C15—C22	113.98 (12)
O1—C1—C12	105.19 (12)	N3—C15—H17	107.3
O1—C1—C13	109.24 (12)	C16—C15—H17	107.3
C12—C1—C13	116.69 (13)	C22—C15—H17	107.3
O1—C1—H1	108.5	O6—C16—N4	123.09 (14)
C12—C1—H1	108.5	O6—C16—C15	121.31 (14)
C13—C1—H1	108.5	N4—C16—C15	115.59 (13)
O2—C2—O1	124.40 (14)	C19—C17—C18	110.89 (14)
O2—C2—C3	123.47 (13)	C19—C17—C3	113.19 (13)
O1—C2—C3	112.08 (12)	C18—C17—C3	109.91 (13)
N1—C3—C2	111.25 (12)	C19—C17—H19	107.5
N1—C3—C17	112.74 (12)	C18—C17—H19	107.5
C2—C3—C17	111.62 (13)	C3—C17—H19	107.5
N1—C3—H2	106.9	C17—C18—H22	109.5
C2—C3—H2	106.9	C17—C18—H21	109.5
C17—C3—H2	106.9	H22—C18—H21	109.5
O3—C4—N1	123.36 (13)	C17—C18—H20	109.5
O3—C4—C5	121.16 (14)	H22—C18—H20	109.5
N1—C4—C5	115.42 (13)	H21—C18—H20	109.5
C20—C5—N2	123.37 (14)	C17—C19—H24	109.5
C20—C5—C4	119.61 (14)	C17—C19—H25	109.5
N2—C5—C4	116.99 (13)	H24—C19—H25	109.5
O4—C6—N2	122.62 (14)	C17—C19—H23	109.5
O4—C6—C7	123.05 (14)	H24—C19—H23	109.5
N2—C6—C7	114.33 (13)	H25—C19—H23	109.5
N4—C7—C8	109.81 (13)	C5—C20—C21	125.28 (16)
N4—C7—C6	109.72 (11)	C5—C20—H26	117.4
C8—C7—C6	108.94 (12)	C21—C20—H26	117.4
N4—C7—H5A	109.4	C20—C21—H27	109.5
C8—C7—H5A	109.4	C20—C21—H29	109.5
C6—C7—H5A	109.4	H27—C21—H29	109.5
C7—C8—S1	116.36 (11)	C20—C21—H28	109.5
C7—C8—H6	108.2	H27—C21—H28	109.5
S1—C8—H6	108.2	H29—C21—H28	109.5
C7—C8—H7	108.2	C23—C22—C24	110.22 (14)
S1—C8—H7	108.2	C23—C22—C15	109.06 (13)
H6—C8—H7	107.4	C24—C22—C15	111.79 (13)
C10—C9—S2	115.36 (11)	C23—C22—H30	108.6
C10—C9—H8	108.4	C24—C22—H30	108.6
S2—C9—H8	108.4	C15—C22—H30	108.6
C10—C9—H9	108.4	C22—C23—H31	109.5
S2—C9—H9	108.4	C22—C23—H32	109.5
H8—C9—H9	107.5	H31—C23—H32	109.5
C11—C10—C9	113.50 (13)	C22—C23—H33	109.5
C11—C10—H10	108.9	H31—C23—H33	109.5
C9—C10—H10	108.9	H32—C23—H33	109.5
C11—C10—H11	108.9	C22—C24—H35	109.5
C9—C10—H11	108.9	C22—C24—H34	109.5
H10—C10—H11	107.7	H35—C24—H34	109.5
C12—C11—C10	125.57 (15)	C22—C24—H36	109.5
C12—C11—H12	117.2	H35—C24—H36	109.5
C10—C11—H12	117.2	H34—C24—H36	109.5
			
C8—S1—S2—C9	−90.18 (7)	S1—S2—C9—C10	65.73 (12)
C2—O1—C1—C12	−162.59 (12)	S2—C9—C10—C11	59.05 (17)
C2—O1—C1—C13	71.42 (15)	C9—C10—C11—C12	−145.87 (15)
C1—O1—C2—O2	12.5 (2)	C10—C11—C12—C1	−172.08 (15)
C1—O1—C2—C3	−170.18 (12)	O1—C1—C12—C11	−146.55 (14)
C4—N1—C3—C2	−135.83 (14)	C13—C1—C12—C11	−25.3 (2)
C4—N1—C3—C17	97.90 (17)	O1—C1—C13—C14	43.45 (16)
O2—C2—C3—N1	−155.37 (14)	C12—C1—C13—C14	−75.63 (16)
O1—C2—C3—N1	27.23 (17)	C15—N3—C14—O5	2.3 (2)
O2—C2—C3—C17	−28.5 (2)	C15—N3—C14—C13	−175.03 (12)
O1—C2—C3—C17	154.12 (12)	C1—C13—C14—O5	−70.20 (18)
C3—N1—C4—O3	0.7 (2)	C1—C13—C14—N3	107.10 (15)
C3—N1—C4—C5	−176.38 (12)	C14—N3—C15—C16	68.10 (16)
C6—N2—C5—C20	131.38 (16)	C14—N3—C15—C22	−165.07 (13)
C6—N2—C5—C4	−50.66 (19)	C7—N4—C16—O6	−4.9 (2)
O3—C4—C5—C20	−46.7 (2)	C7—N4—C16—C15	176.20 (12)
N1—C4—C5—C20	130.44 (16)	N3—C15—C16—O6	−152.01 (13)
O3—C4—C5—N2	135.23 (15)	C22—C15—C16—O6	83.37 (17)
N1—C4—C5—N2	−47.60 (18)	N3—C15—C16—N4	26.90 (17)
C5—N2—C6—O4	−3.8 (2)	C22—C15—C16—N4	−97.72 (15)
C5—N2—C6—C7	176.31 (12)	N1—C3—C17—C19	61.93 (18)
C16—N4—C7—C8	−160.22 (13)	C2—C3—C17—C19	−64.14 (16)
C16—N4—C7—C6	80.06 (16)	N1—C3—C17—C18	−62.66 (17)
O4—C6—C7—N4	114.64 (16)	C2—C3—C17—C18	171.27 (12)
N2—C6—C7—N4	−65.46 (16)	N2—C5—C20—C21	−3.5 (3)
O4—C6—C7—C8	−5.61 (19)	C4—C5—C20—C21	178.62 (16)
N2—C6—C7—C8	174.30 (12)	N3—C15—C22—C23	66.98 (16)
N4—C7—C8—S1	68.66 (14)	C16—C15—C22—C23	−168.10 (12)
C6—C7—C8—S1	−171.15 (10)	N3—C15—C22—C24	−170.88 (13)
S2—S1—C8—C7	−69.44 (12)	C16—C15—C22—C24	−45.97 (19)

### Hydrogen-bond geometry (Å, º)

**Table:**

*D*—H···*A*	*D*—H	H···*A*	*D*···*A*	*D*—H···*A*
N1—H3···O5	0.84 (2)	2.27 (2)	3.0123 (18)	147.1 (17)
N2—H4···O6	0.82 (2)	2.05 (2)	2.7867 (18)	149.1 (18)
N3—H16···O3^i^	0.87 (2)	2.18 (2)	3.0449 (17)	175.9 (18)

Symmetry code: (i) −*x*+1, *y*−1/2, −*z*.
